# Estimating recent trends in alcohol sales in the United Kingdom from alcohol duty revenue

**DOI:** 10.1111/add.70109

**Published:** 2025-06-19

**Authors:** Colin Angus, Jonas Schöley

**Affiliations:** ^1^ Sheffield Addictions Research Group University of Sheffield Sheffield UK; ^2^ Laboratory of Population Health Max Planck Institute for Demographic Research Rostock Germany

**Keywords:** alcohol, alcohol consumption, alcohol taxes, counterfactual modelling, COVID‐19, public health

## Abstract

**Background and Aims:**

The onset of the COVID‐19 pandemic led to significant changes in individual‐level alcohol consumption and a sharp increase in heavy drinking in the United Kingdom (UK). More recently, high rates of inflation, the resulting ‘cost of living crisis’ and reforms to alcohol taxation have affected the affordability of alcohol, but little is understood about how these changes have impacted on alcohol sales and consumption. We aimed to measure recent trends in alcohol sales by assessing changes in alcohol duty revenue collected by the UK government since 2020.

**Methods:**

We used published data on UK alcohol duty revenue to model trends from 2010 to 2019. We forecasted these trends through to January 2025 using a novel statistical approach and compared these forecasts with observed receipts. Measurements included monthly inflation‐adjusted alcohol duty receipts received by the UK Treasury in pounds sterling for beer, cider, spirits and wine.

**Results:**

During the pandemic, alcohol duty receipts fell during lockdowns and rose as restrictions were subsequently lifted. Since 2022 alcohol duty receipts have been consistently statistically significantly below the historical trend, with a cumulative deficit of £10.3bn (−12.3%). This has not been uniform across beverage types, with a gradually increasing deficit in wine receipts and a comparable deficit in spirits receipts that began sharply in late 2022 compared with smaller deficits for beer and cider. The reforms to the alcohol duty system in August 2023 do not appear to have substantially affected these trends.

**Conclusions:**

The ‘cost of living crisis’ in 2022/2023 appears to have been associated with a fall in alcohol sales in the United Kingdom relative to the pre‐pandemic trend. The magnitude of this fall differs by beverage type, indicating that wine and spirits drinkers may have changed their behaviour more than beer and cider drinkers.

## INTRODUCTION

The harms associated with alcohol consumption place a substantial burden on society, with total societal costs estimated to exceed £27billion per year in England [1]. The onset of the coronavirus disease 2019 (COVID‐19) pandemic in 2020 was associated with marked changes in alcohol consumption for many individuals, with multiple studies finding a polarisation in drinking, with heavier drinkers drinking more and moderate drinkers consuming less alcohol, or giving up entirely [2, 3, 4, 5]. Alongside these changes, the United Kingdom (UK) has seen a sharp increase in deaths from wholly alcohol‐attributable causes, rising by 38.4% between 2019 and 2023 to their highest level on record [6].

Beyond the pandemic, inflation rates in the United Kingdom began to rise in 2021, reaching a 40‐year high in October 2022, leading to a so‐called ‘cost‐of‐living crisis’ as the costs of goods and energy rose faster than incomes [7]. Further, in August 2023, the UK Government introduced reforms to alcohol taxation, designed with the explicit goal of improving public health. Before these reforms, beer and spirits were taxed on the basis of their alcohol content, whereas wine and cider were taxed on the basis of the volume of product sold. The reforms changed the basis for taxation for wine and cider so that all alcohol products are now taxed on the basis of their alcohol content, with duty rates rising with alcoholic strength in line with recommendations from the World Health Organization [8, 9]. Although these reforms were designed to be broadly revenue neutral for government, at the same time as their implementation alcohol duty rates were increased by 10.1% across all beverage types, in line with inflation [8], the largest single increase in alcohol taxes for over 40 years.

To date, studies on changes in alcohol consumption since the start of 2020 have used individual‐level survey data, which may be subject to self‐report bias, and focused on changes during COVID restrictions in the early phase of the pandemic. Little is known about how consumption may have changed in the subsequent period during the cost‐of‐living crisis or in response to the alcohol duty reforms.

Alcohol duties brought in £12.1 billion in revenue to the UK Government in 2019, a figure that had been rising steadily, even after adjusting for inflation, for at least 20 years [10]. To understand the impact of events since 2019 on alcohol consumption and the potential implications for future rates of alcohol harms, we examined UK government data on alcohol duty revenues, comparing revenues since 2020 to the pre‐pandemic trend. Duty revenues represent a strong proxy measure for the volume of alcohol sold in the United Kingdom as the majority of alcohol is taxed on the basis of its alcohol content.

## METHODS

To detect unusual alcohol consumption patterns since 2020 we estimate the deviation between observed and expected UK alcohol duty receipts. We used monthly data on UK alcohol duty receipts published by His Majesty’s Revenue and Customs for the period from January 2010 to January 2025 [11]. Data is published separately for duty receipts from beer, cider, spirits and wine. All values were inflated to January 2025 prices using the Retail Prices Index, the inflation measure used to uprate duty rates annually [12]. Deviations in receipts since 2020 relative to pre‐pandemic trends, by beverage type, were assessed using a methodological approach that has previously been applied to estimate cause‐specific excess mortality during the pandemic [13]. The calculation of expected receipts involved a two‐stage process.

First, using data from January 2010 to December 2019, we modelled the expected total monthly inflation adjusted duty revenues at time *t*, denoted as ${\hat{y}}_{t}$, as a log‐linear function of year and month via

(1) $$\log\left({\hat{y}}_{t}\right)=\beta_{0}+\beta_{1}{\left(\text{months since}\;\mathrm{Jan}\;2010\right)}_{t}+\beta_{\text{month of year}},$$

where $\beta_{1}$ is the slope of a log‐linear long‐term trend and $\beta_{\text{month}\;\text{of}\;\text{year}}$ is a month‐specific fixed effect, to account for seasonality.

Second, we fit a compositional regression to estimate the expected proportion of total duty receipts that come from each beverage type i at time t, denoted $\;{\hat{\pi }}_{t}^{i},$ using the isometric log‐ratio transformation, which maps compositional data to an unrestricted real valued domain, suitable for the application of standard statistical approaches, while preserving the relative relationship between the dimensions (in this case beverage types) [14].

(2) $$\mathrm{ilr}\left({\hat{\pi }}_{t}^{i}\right)=\beta_{0}^{i}+\beta_{1}^{i}{\left(\text{months since}\;\mathrm{Jan}\;2010\right)}_{t}+\beta_{\text{month of year}}^{i}.$$

We can then estimate the expected receipts for any beverage type i at time t as

(3) $${\hat{y}}_{t}^{i}={\hat{y}}_{t}{\hat{\pi }}_{t}^{i}.$$

Essentially, equation 1 models the overall trend in alcohol duty revenue, equation 2 models the trends in the proportion of revenue coming from each beverage type and equation 3 allows us to combine the outcomes of these two models to derive the expected receipts for any beverage type at any time point.

A multivariate normal distribution of errors in the predicted duty revenues, correlated across beverage types, has been estimated from the residuals of the fitting data on the log‐ratio scale. To quantify the uncertainty around our estimates we perform a residual bootstrap from this distribution and used it to construct the prediction intervals around the revenue expectations.

The compositional approach was chosen to ensure coherency between expected duty revenues on both the beverage specific and the total level [15]. Duty revenues by beverage sum up to total revenues and prediction intervals, being derived from correlated errors, have good calibration on both the beverage specific level of analysis as well as the total level (Table S1).

Central assumptions of the model are the multivariate normality of the predictive distribution of revenue on the log‐ratio scale and the linearity of the long‐term revenue trends. The good model calibration shows that the first assumption is unproblematic and the linear revenue trend has been shown to perform better than a quadratic specification when forecasting revenue (Table S2).

Based on the fitted models we predict expected receipts by beverage type for the months following January 2020. Deviations from the expectation are the given by the simple difference between observed and expected duty revenues.

See the supporting information for diagnostic plots of model fit (Figures S1 and S2). An inspection of long‐term trends in duty revenue by beverage type (Figure S3) suggests the possibility that trends in wine revenues may have changed in early 2018. We, therefore, undertook a sensitivity analysis where we used data up to December 2017 as the training data and assessed deviation from this trend from January 2018 onward. All analyses were performed using R statistical software. Full code to replicate the analysis can be found at https://github.com/VictimOfMaths/Experiments/blob/master/HMRCReceiptsAnalysis.R. This analysis was not pre‐registered and should, therefore, be considered exploratory.

## RESULTS

**FIGURE 1:**
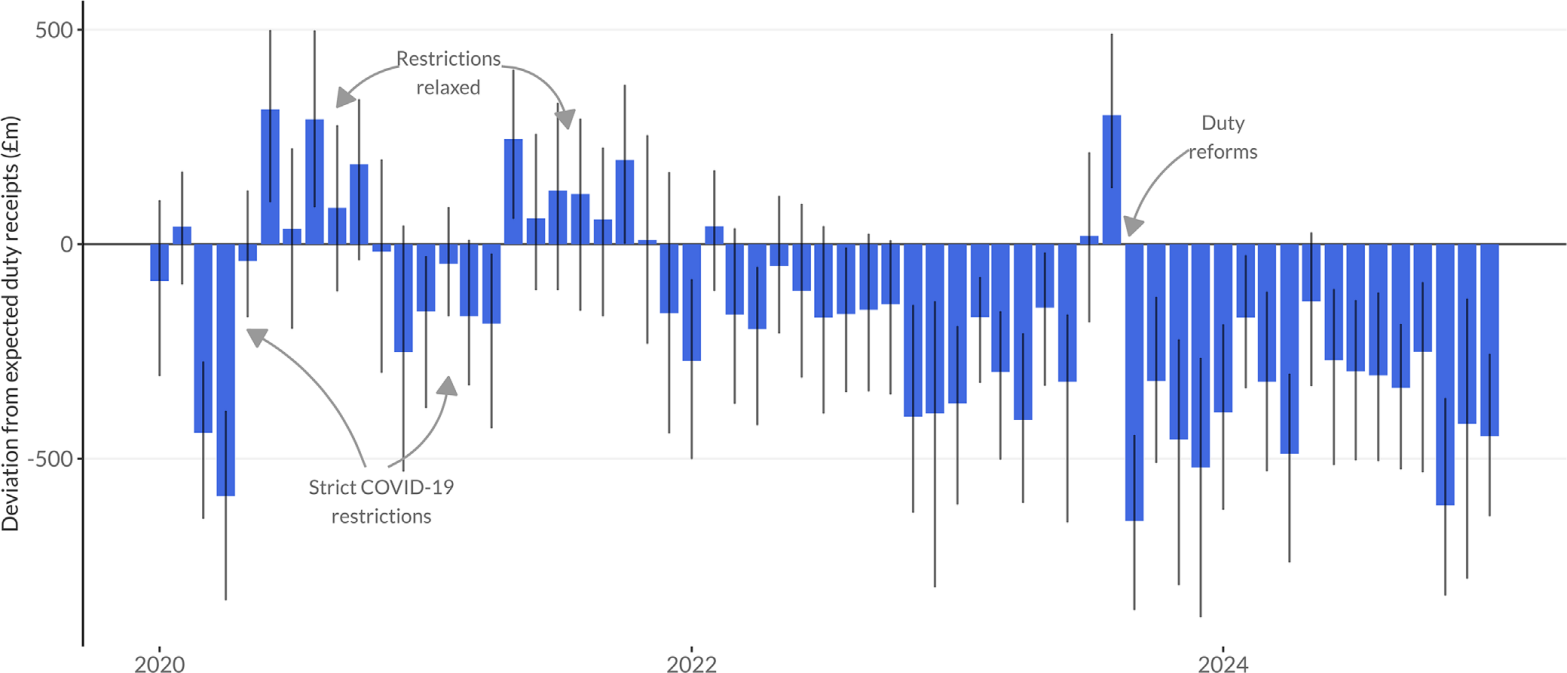
Deviation from expected monthly duty revenue in the United Kingdom since January 2020 with 95% prediction intervals.

**FIGURE 2:**
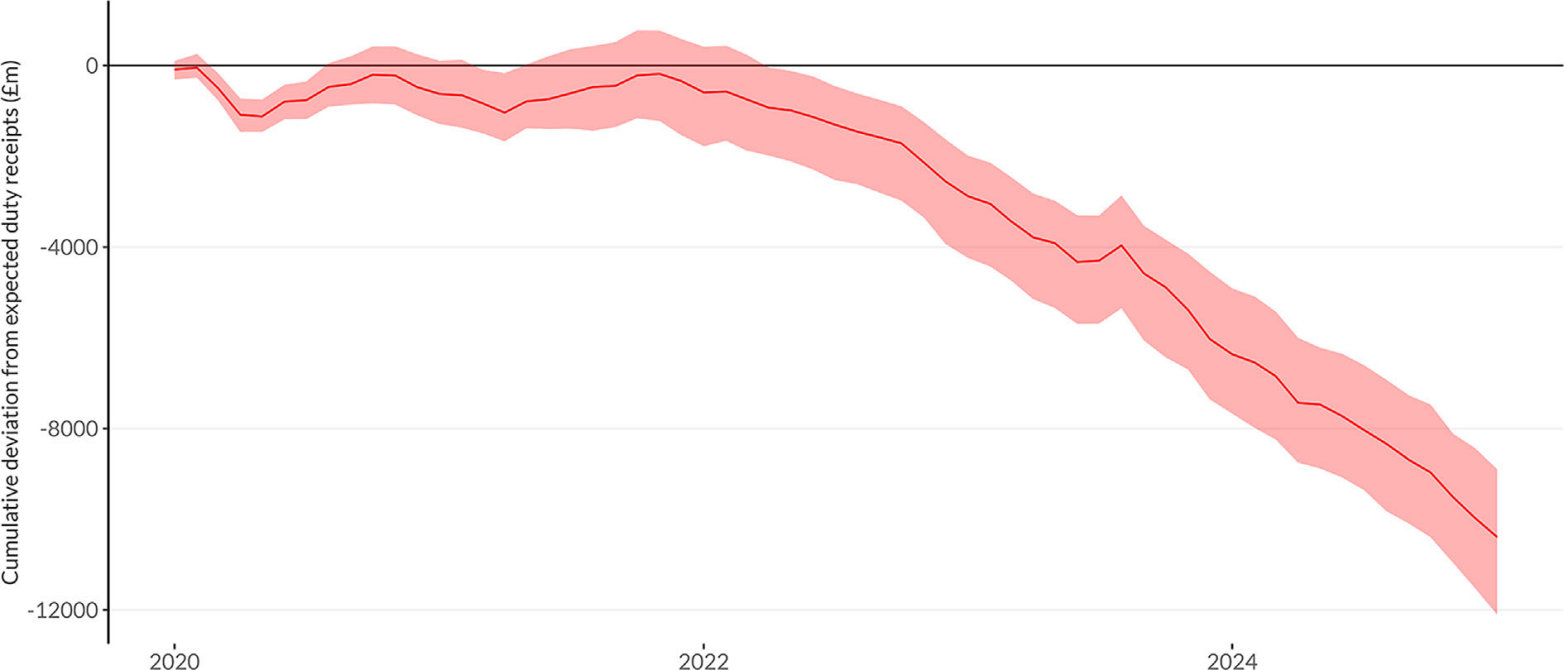
Cumulative deviation from expected duty revenue in the United Kingdom since January 2020 with 95% prediction intervals.

Figure 1 shows the monthly deviation from expected duty revenue collected by UK Government since January 2020. This illustrates that duty receipts fell sharply in March and April 2020, when pandemic lockdown measures were first introduced, but this was offset by a corresponding increase in revenues in the summer of 2020 as restrictions were lifted. This pattern was repeated in late 2020/early 2021 when lockdowns were reintroduced, and subsequently relaxed in spring 2021, albeit with a smaller deviation from expected receipts than the first lockdown. Figure 2 shows the cumulative difference between observed and expected receipts since January 2020, illustrating that the net impact of these changes in duty receipts across 2020 and 2021 was not significantly different from zero. See Figures S4 and S5 for equivalent figures showing the relative deviations from expected receipts.

Since the start of 2022 when inflation rates began to rise, duty receipts have consistently been significantly below the levels that would have been expected based on pre‐pandemic trends. This deficit appears to widen in November 2022, when inflation rates peaked and has remained at this higher level through into early 2024. The only deviation from this pattern was in August 2023, when duty receipts rose sharply. This almost certainly reflects alcohol producers choosing to pay duty earlier than they usually would on products stored in their warehouses in advance of the increases in alcohol duty that came into force that month, a process known as ‘forestalling’. Overall, since January 2020 alcohol duty receipts have been £10.3 billion lower than expected, a reduction of 12.3%.

**FIGURE 3:**
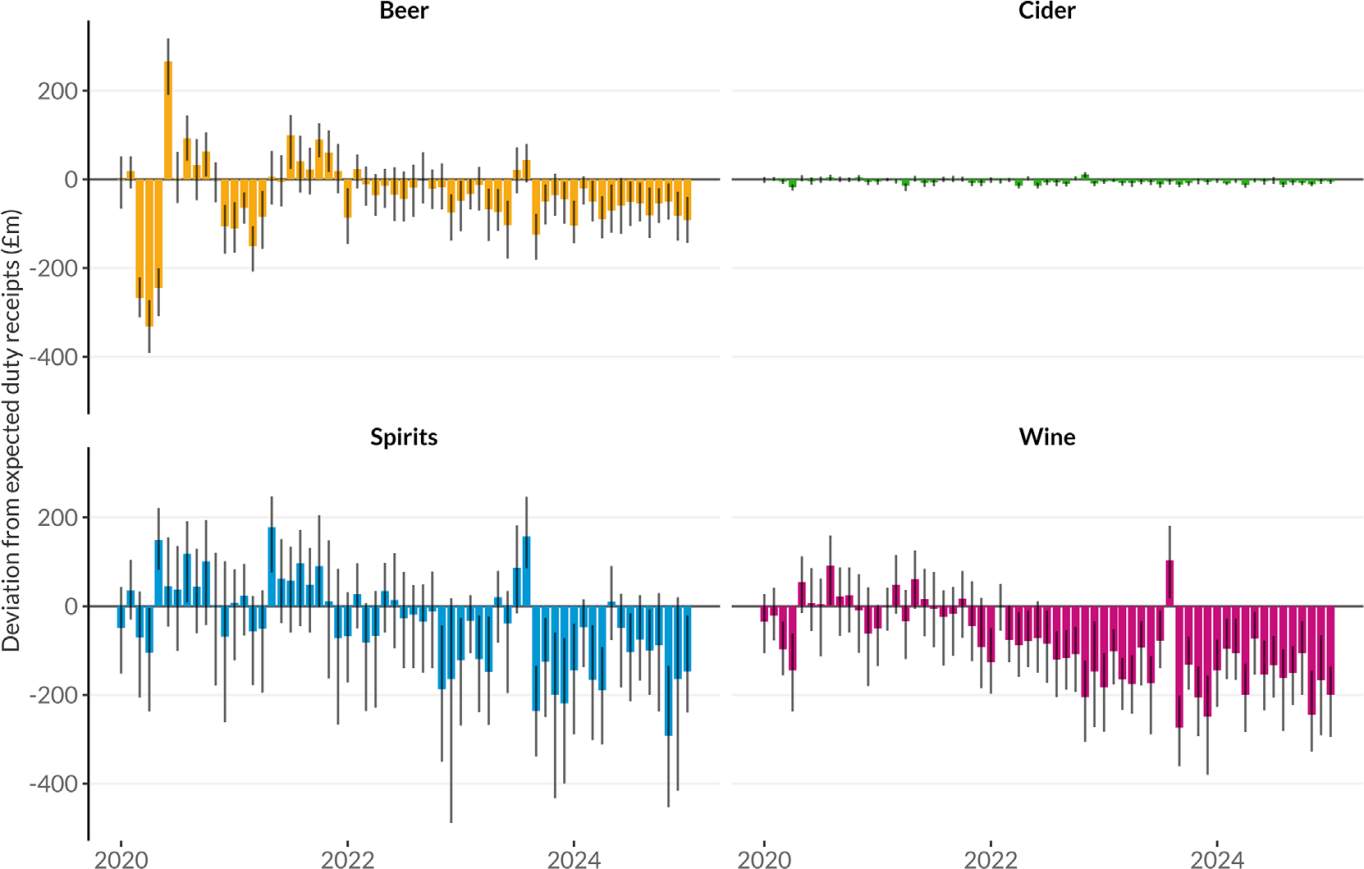
Deviation, by beverage type, from expected monthly duty revenue in the United Kingdom since January 2020 with 95% prediction intervals.

**FIGURE 4:**
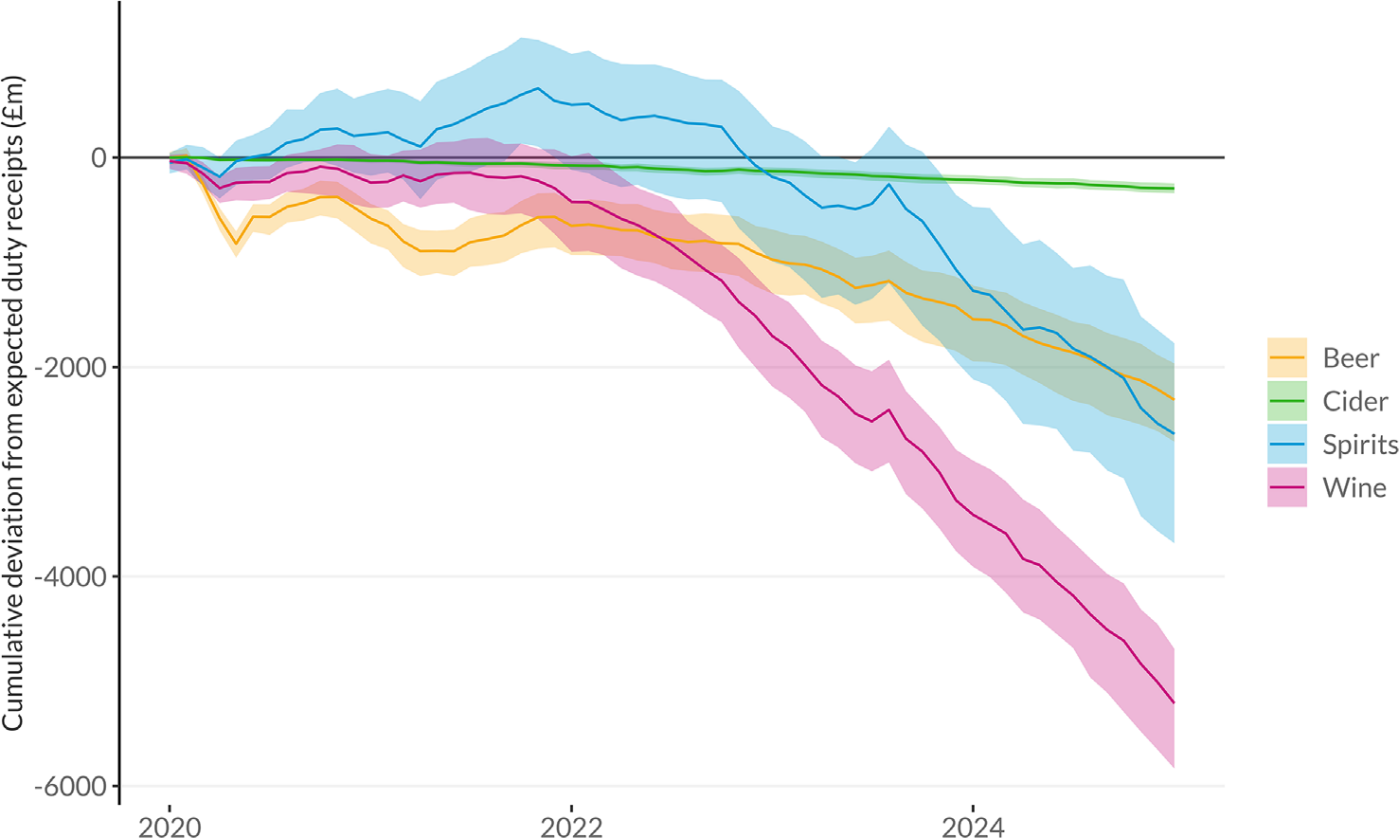
Cumulative deviation, by beverage type, from expected duty revenue in the United Kingdom since January 2020 with 95% prediction intervals.

Figure 3 presents the monthly deviation between observed and expected receipts since January 2020 by beverage type, with the equivalent cumulative difference shown in Figure 4. These show larger falls in duty receipts for beer during the COVID lockdowns, and larger increases in receipts for spirits in their immediate aftermath. Beer also shows comparatively smaller falls relative to the pre‐pandemic trend since 2022 compared to wine and spirits. For wine, receipts have been consistently significantly below expected levels since late 2021, with this deficit increasing gradually throughout 2022 to approximately £150 m per month (−27%). In contrast, spirits receipts were at expected levels for most of 2022 until November, when a significant deficit appeared that has remained broadly stable ever since at approximately £100 m per month (−20%). For beers, wines and spirits, the fall in duty revenues stopped briefly in August 2023, with revenues in that month significantly higher than expected. This corresponds to the final month in which alcohol producers could clear products for sale (i.e. pay the duty on them) before the duty reforms and 10.1% increase in duty rates. However, Figure 4 illustrates that this represented a temporary outlier in the underlying trend rather than a shift in the direction of that trend. Equivalent relative versions of these Figures S6 and S7 can be found in the Supporting information.

In a sensitivity analysis using only pre‐2018 training data, to assess whether trends in receipts were already deviating from prior trends before the COVID pandemic, our results are broadly similar, with no clear indication of a change in overall duty revenue trends before 2020 (see Figures S8–S11 in the Supporting information). Beverage‐specific results suggest that beer receipts may have been rising above pre‐2018 trends and wine receipts below pre‐2018 trends before any impact of the pandemic, however, the changes in trends during the pandemic and the cost‐of‐living crisis identified in our primary analysis remain evident.

## DISCUSSION

Our analysis shows that, relative to pre‐pandemic trends, government revenue from alcohol duties was relatively unchanged overall across 2020 and 2021, although revenues fell during periods of lockdown with corresponding increases as restrictions were lifted. The falls in revenue were greatest for beer, most likely because of the fact that pubs were closed during lockdowns and beer is consumed disproportionately in the on‐trade [16]. Meanwhile spirits revenues increased above expected levels over this period, with the largest rises in period when restrictions were less stringent.

More recently, overall alcohol duty revenues since early 2022 have fallen relative to the pre‐pandemic trend, with a cumulative deficit of £10.3 billion overall, a fall of 12.3%. This suggests that alcohol sales are likely to have fallen, on average, during the cost‐of‐living crisis. Previous studies have shown two key mechanisms at play in relation to alcohol consumption during economic downturns: reductions in consumption as individuals cut back on non‐essential spending in the face of financial pressure and increases in consumption in response to increased stress driven by economic challenges [17]. Recent data suggests 7 million adults in Great Britain (1 in 8) reported experiencing financial hardship during 2022 and/or early 2023 and that this was strongly associated with higher rates of psychological distress [18]. Our analysis suggests a greater degree of cutting back than drinking to cope with stress, at least at the population level, although there may still be important differences between individuals. The potential for this is highlighted by the fact that by far the largest cumulative reduction in duty revenues compared to expectation is in wine, which is more commonly drunk by those in higher socio‐economic groups [19, 20]. This is somewhat contrary to expectations, as it is lower socio‐economic groups who have been most affected by the cost‐of‐living crisis [7]. It is also surprising that wine receipts have fallen gradually below expected levels since late 2021, whereas spirits, and to a lesser extent beer, receipts only fell below expected levels in November 2022, suggesting that there may be different underlying causes for these trends, with increased bureaucracy associated with the import of alcohol following Brexit potentially impacting the wine industry, and wine prices, more than other products. Further research is needed to examine individual changes in alcohol consumption over this period to understand the impact of these economic challenges and the resulting implications for alcohol harms. This is particularly important given that we would expect falling alcohol sales, implying a fall in per capita consumption, to be associated with reductions in alcohol harm. Yet alcohol‐specific deaths in the United Kingdom have continued to rise, suggesting that not all groups in the population are drinking less and some may even be drinking more.

Finally, our results show clear evidence of ‘forestalling’ or bringing forward of the payment of alcohol duties by the alcohol industry in relation to the August 2023 duty reforms, with large volumes of alcohol apparently being cleared for sale immediately before the reforms that would likely otherwise have been cleared in the months thereafter. However, there is no clear evidence of a longer‐term impact of the reforms on duty receipts, rather than a continuation of pre‐reform trends, contrary to claims made by the alcohol industry [21].

Our analysis used robust data and a novel approach to examine trends in alcohol duty receipts up to October 2024, however, there are several important limitations. Most importantly, although there is a strong correlation between duty revenues and alcohol sales volumes, receipts are not a direct measure of alcohol sales or consumption, and alternative national‐level estimates of alcohol consumption or sales are not readily available for comparison. Although spirits and beer are taxed directly on the basis of alcohol content, wine and cider duty before the duty reforms was based on product volume. As a result, if consumers had shifted from higher‐ to lower‐ strength wines, or vice versa, this would not be captured in duty receipts. Further, these figures relate to alcohol cleared for sale, not alcohol sold and, therefore, do not account for potential stockpiling by either alcohol producers/retailers or by consumers. It is also possible that differential price changes between beverage types may have driven shifts in sales between products, although beverage‐specific inflation indices published by the UK Government do not suggest large deviations in prices over time by beverage (see Figure S12). Finally, we have not accounted for increases in tourism as COVID restrictions were relaxed [22], both in terms of UK residents purchasing alcohol abroad and foreign residents purchasing alcohol in the United Kingdom, although the combination of these effects on overall alcohol sales is likely to be small [23].

Overall our findings suggest little overall change in alcohol sales in the United Kingdom during the COVID‐19 pandemic relative to pre‐pandemic trends, but a clear fall in sales during the cost‐of‐living crisis driven initially by wine and, more recently, spirits. We find little evidence that the duty reforms have impacted on pre‐reform trends.

## AUTHOR CONTRIBUTIONS

**Colin Angus:** Data curation (lead); formal analysis (equal); methodology (supporting); visualization (lead); writing—original draft (lead); writing—review and editing (equal). **Jonas Schöley:** Conceptualization (equal); formal analysis (equal); methodology (lead); visualization (supporting); writing—review and editing (equal).

## DECLARATION OF INTERESTS

None.

## Supporting information


**Figure S1.** Modelled (red line with 95% prediction intervals) and observed (black circles) alcohol duty receipts. Vertical dashed line represents the end of the training data period.
**Figure S2.** Modelled (red line with 95% prediction intervals) and observed (black circles) alcohol duty receipts by beverage type. Vertical dashed line represents the end of the training data period.
**Figure S3.** Raw alcohol duty receipts (12‐month rolling average) 2000–2025.
**Figure S12.** Changes in CPI inflation since January 2010 for alcohol and all goods and services.
**Figure S4.** Relative deviation from expected duty receipts in the UK since January 2020 with 95% prediction intervals.
**Figure S5.** Relative cumulative deviation from expected alcohol duty revenue in the UK since January 2020 with 95% prediction interval.
**Figure S6.** Relative deviation from expected duty receipts in the UK by beverage type since January 2020 with 95% prediction intervals.
**Figure S7.** Relative cumulative deviation from expected alcohol duty revenue in the UK by beverage type since January 2020 with 95% prediction interval.
**Figure S8.** Excess monthly duty revenue in the UK since January 2018 with 95% prediction intervals.
**Figure S9.** Cumulative excess duty revenue since January 2018 with 95% prediction interval.
**Figure S10.** Excess monthly duty revenue in the UK by beverage type since January 2018 with 95% prediction intervals.
**Figure S11.** Cumulative beverage‐specific excess duty revenue since January 2018 with 95% prediction intervals.
**Table S1.** Coverage of monthly duty revenue 95% prediction intervals over the training period.
**Table S2.** Comparison of model prediction errors in training dataset under alternative assumptions about the underlying time trend.

## Data Availability

All data is publicly available. R code to replicate our analysis is available at https://github.com/VictimOfMaths/Experiments/blob/master/HMRCReceiptsAnalysis.R.
