# Supplementary material for: Estimating recent trends in alcohol sales in the United Kingdom from alcohol duty revenue
Source: Addiction. 2025 Jun 19;120(10):2134–40. doi: 10.1111/add.70109 (PMC12426364; doi:10.1111/add.70109)
Supplement: Supplementary file 1 — Figure S1. Modelled (red line with 95% prediction intervals) and observed (black circles) alcohol duty receipts. Vertical dashed line represents the end of the training data period. Figure S2. Modelled (red line with 95% prediction intervals) and observed (black circles) alcohol duty receipts by beverage type. Vertical dashed line represents the end of the training data period. Figure S3. Raw alcohol duty receipts (12‐month rolling average) 2000–2025. Figure S12. Changes in CPI inflation since January 2010 for alcohol and all goods and services. Figure S4. Relative deviation from expected duty receipts in the UK since January 2020 with 95% prediction intervals. Figure S5. Relative cumulative deviation from expected alcohol duty revenue in the UK since January 2020 with 95% prediction interval. Figure S6. Relative deviation from expected duty receipts in the UK by beverage type since January 2020 with 95% prediction intervals. Figure S7. Relative cumulative deviation from expected alcohol duty revenue in the UK by beverage type since January 2020 with 95% prediction interval. Figure S8. Excess monthly duty revenue in the UK since January 2018 with 95% prediction intervals. Figure S9. Cumulative excess duty revenue since January 2018 with 95% prediction interval. Figure S10. Excess monthly duty revenue in the UK by beverage type since January 2018 with 95% prediction intervals. Figure S11. Cumulative beverage‐specific excess duty revenue since January 2018 with 95% prediction intervals. Table S1. Coverage of monthly duty revenue 95% prediction intervals over the training period. Table S2. Comparison of model prediction errors in training dataset under alternative assumptions about the underlying time trend. [file ADD-120-2134-s001.docx]

**Estimating recent trends in UK alcohol sales from alcohol duty revenue**

**Online Supplementary Material**


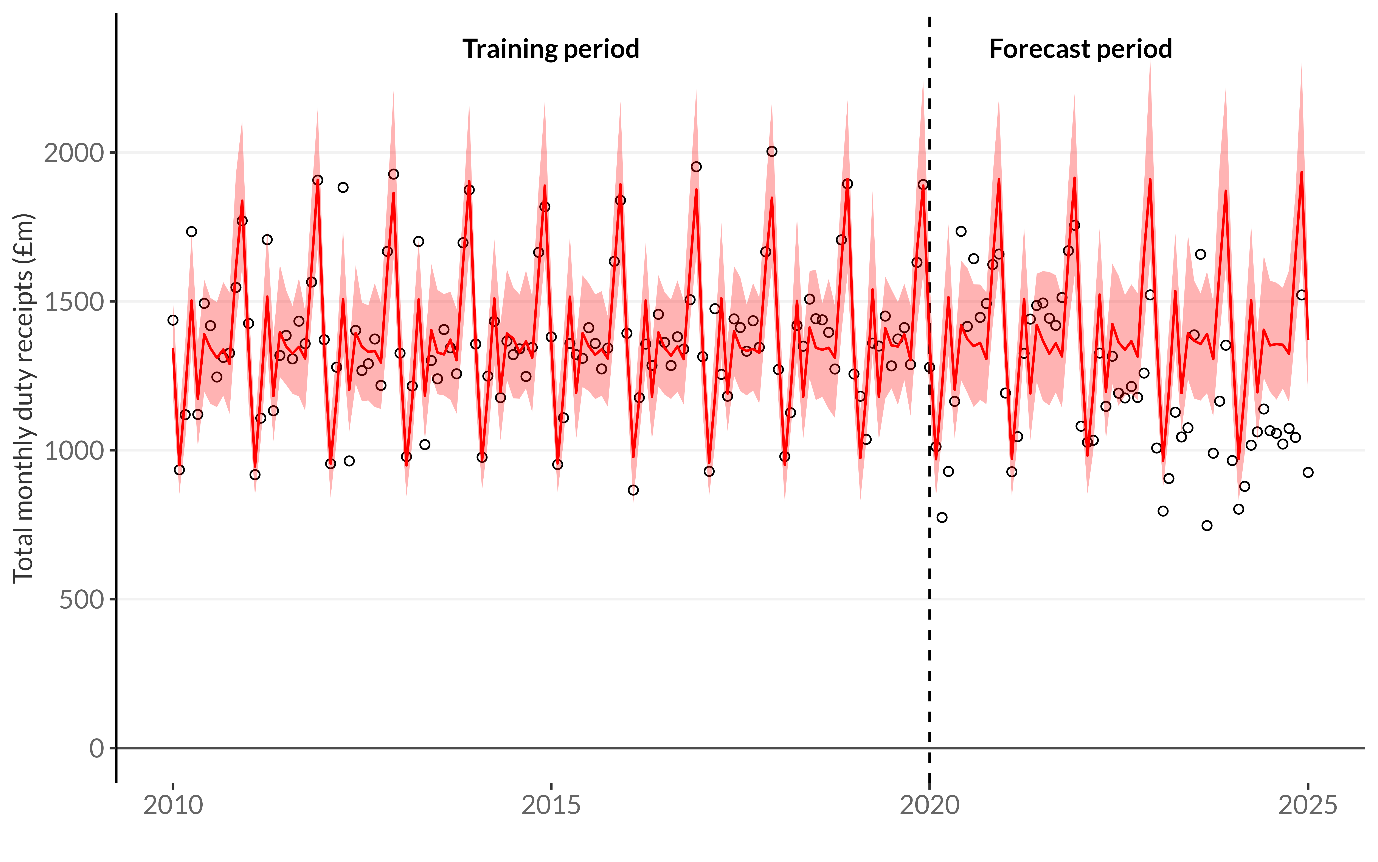


Figure A1 - Modelled (red line with 95% prediction intervals) and observed (black circles) alcohol duty receipts. Vertical dashed line represents the end of the training data period


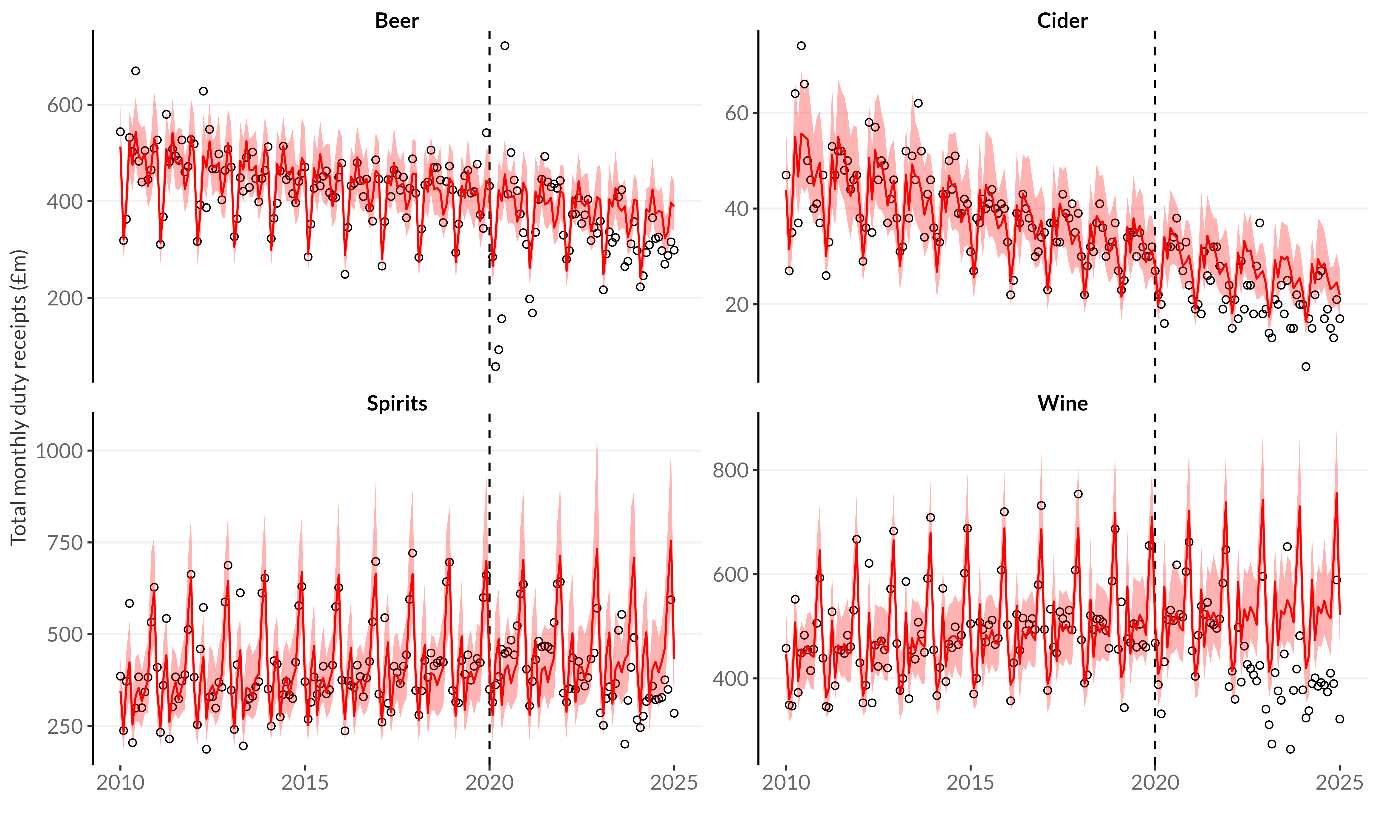


Figure A2 - Modelled (red line with 95% prediction intervals) and observed (black circles) alcohol duty receipts by beverage type. Vertical dashed line represents the end of the training data period

| **Revenue type** | **Coverage** |
| --- | --- |
| Beer | 95% |
| Cider | 90.8% |
| Spirits | 90.8% |
| Wine | 94.2% |
| Total | 92.5% |

Table A1 – Coverage of monthly duty revenue 95% prediction intervals over the training period.

| **Model specification** | **Mean Average Percentage Error** |
| --- | --- |
| Linear trend | 5.35% |
| Quadratic trend | 5.45% |

Table A2 – Comparison of model prediction errors in training dataset under alternative assumptions about the underlying time trend


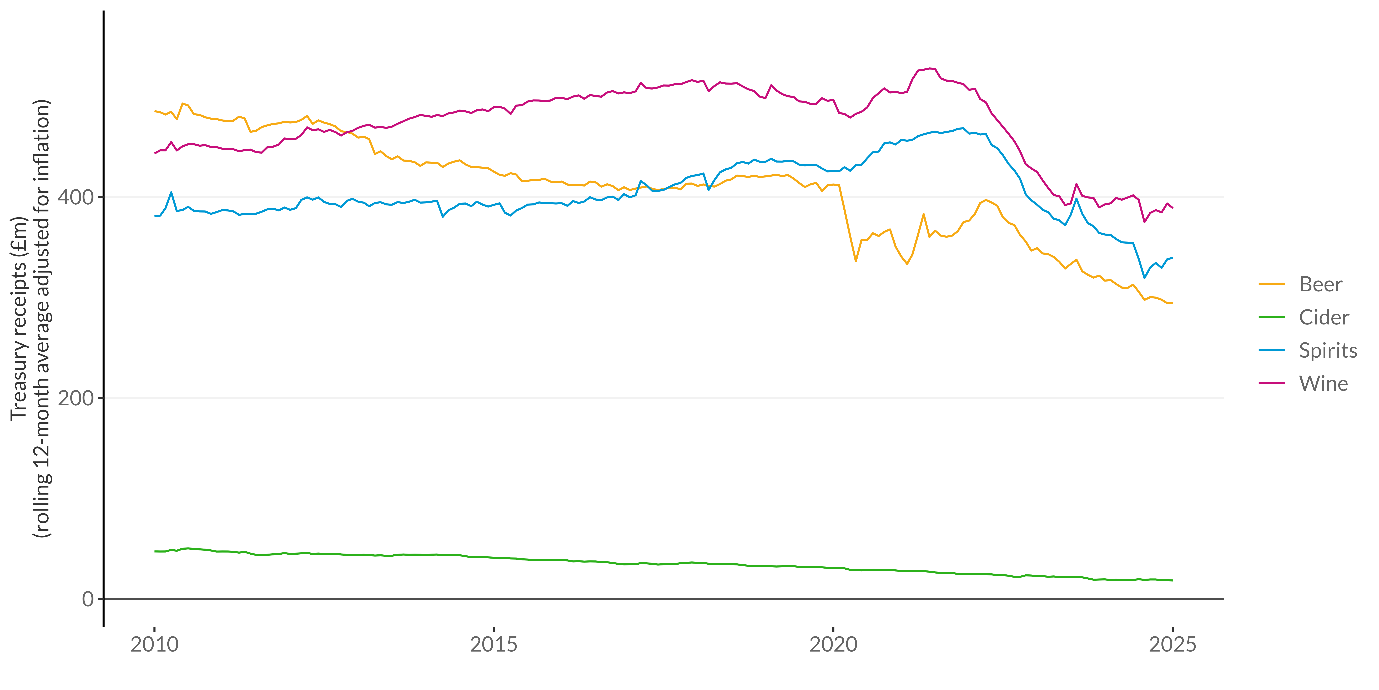


Figure A3 - Raw alcohol duty receipts (12-month rolling average) 2000-2025


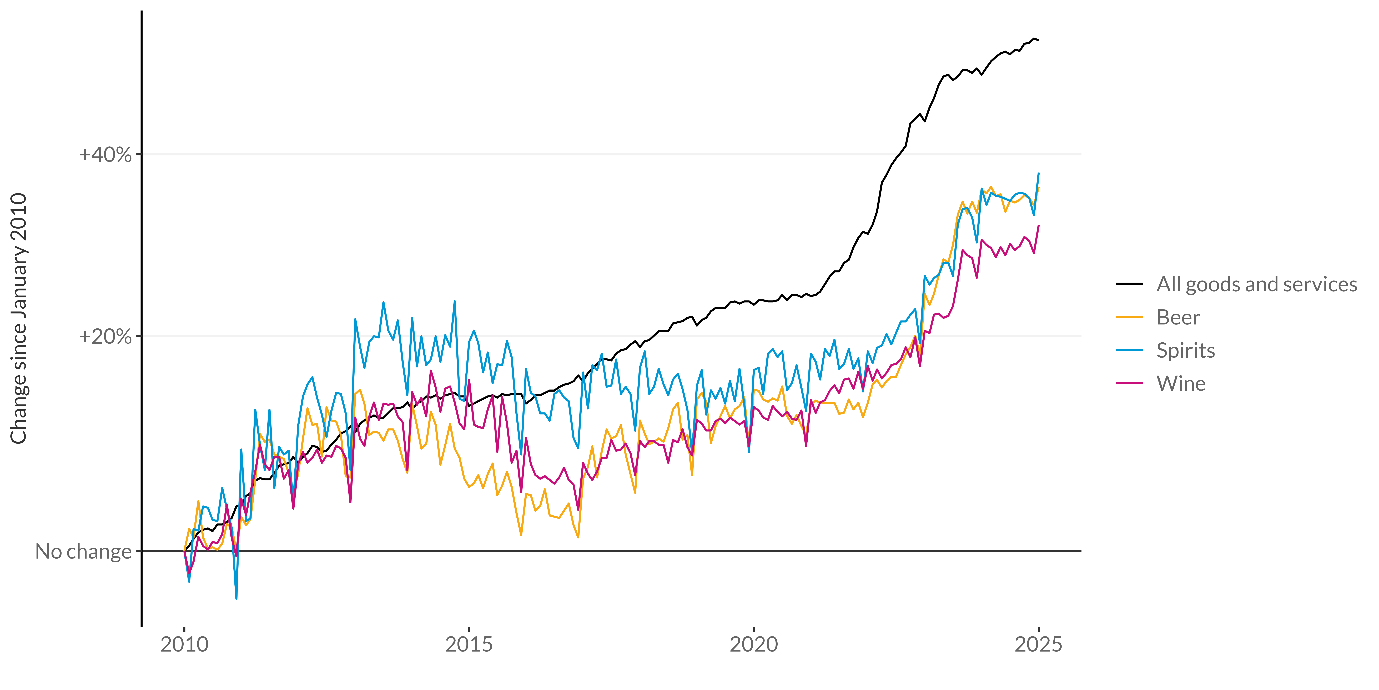


Figure A4 - Changes in CPI inflation since January 2010 for alcohol and all goods and services

**Relative model outcomes**


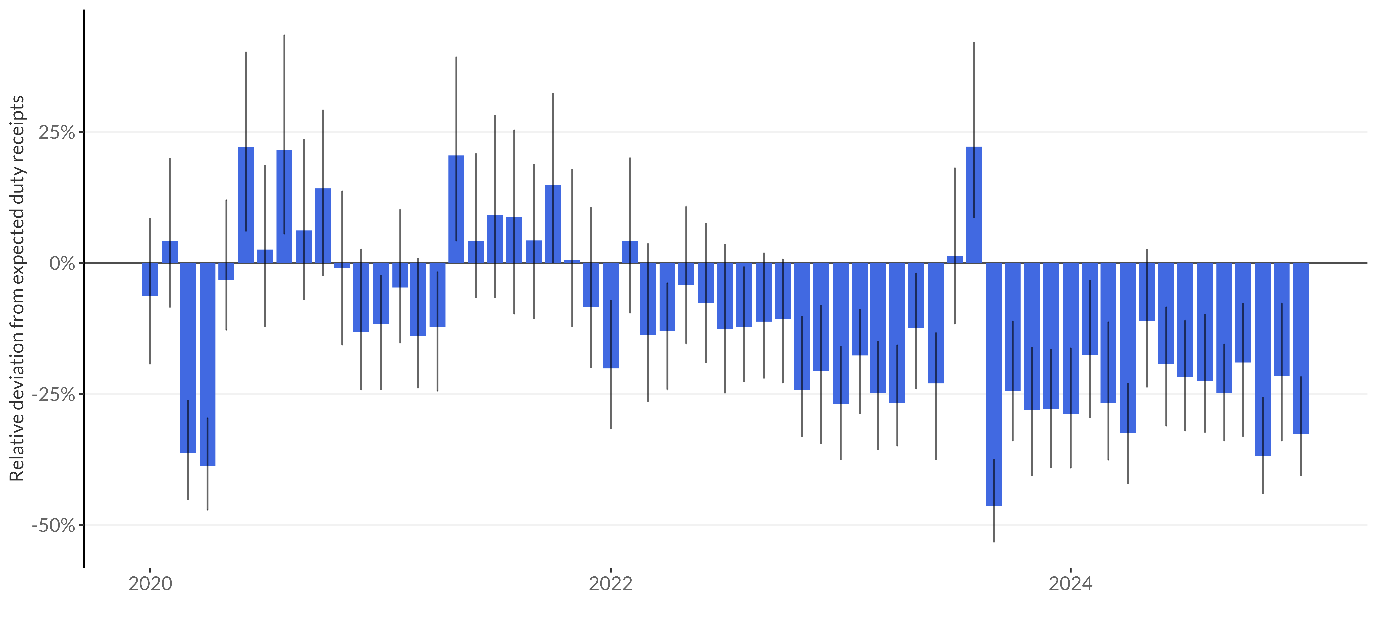


Figure A5 – Relative deviation from expected duty receipts in the UK since January 2020 with 95% prediction intervals


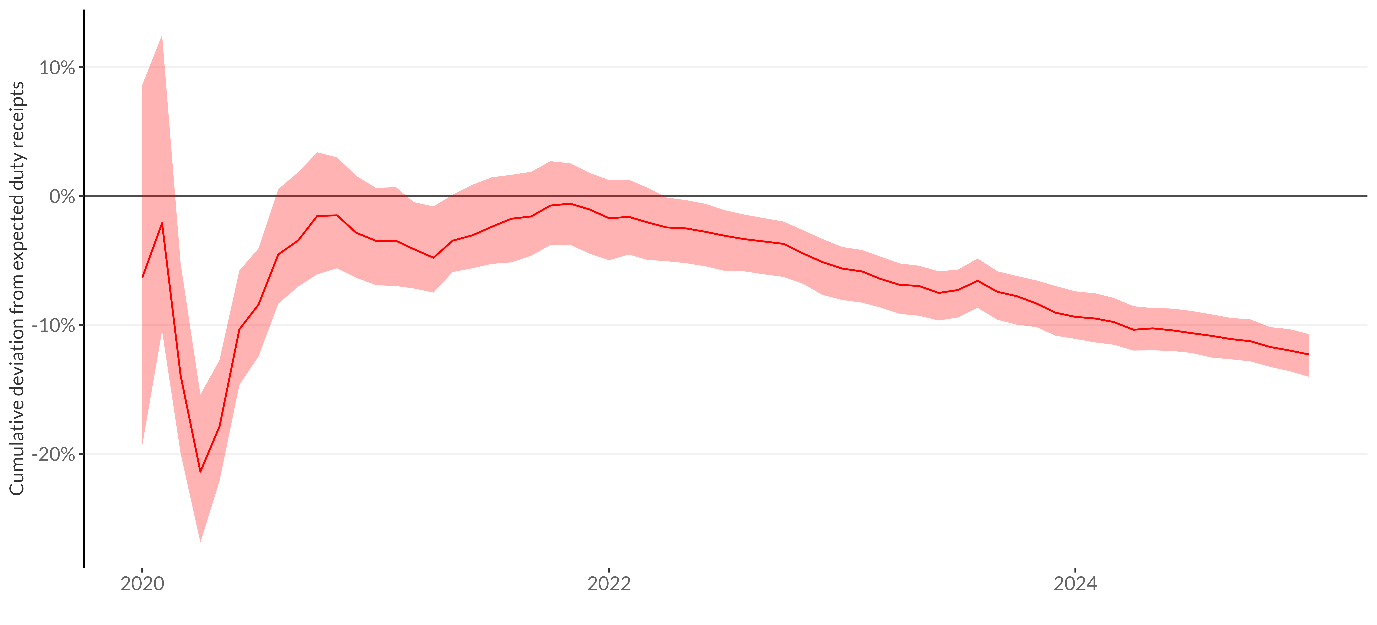


Figure A6 – Relative cumulative deviation from expected alcohol duty revenue in the UK since January 2020 with 95% prediction interval

**
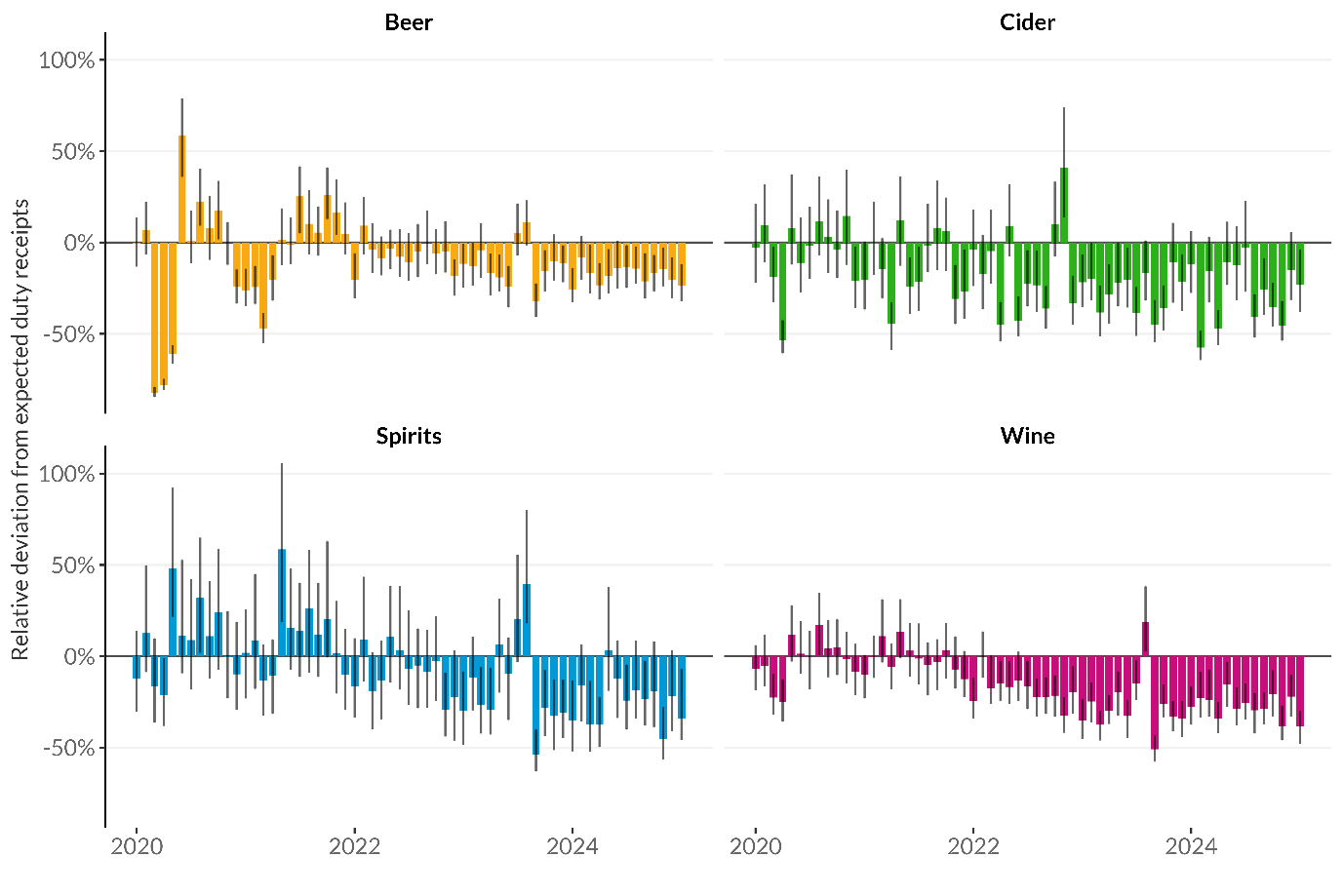
**

Figure A7 - Relative deviation from expected duty receipts in the UK by beverage type since January 2020 with 95% prediction intervals

**
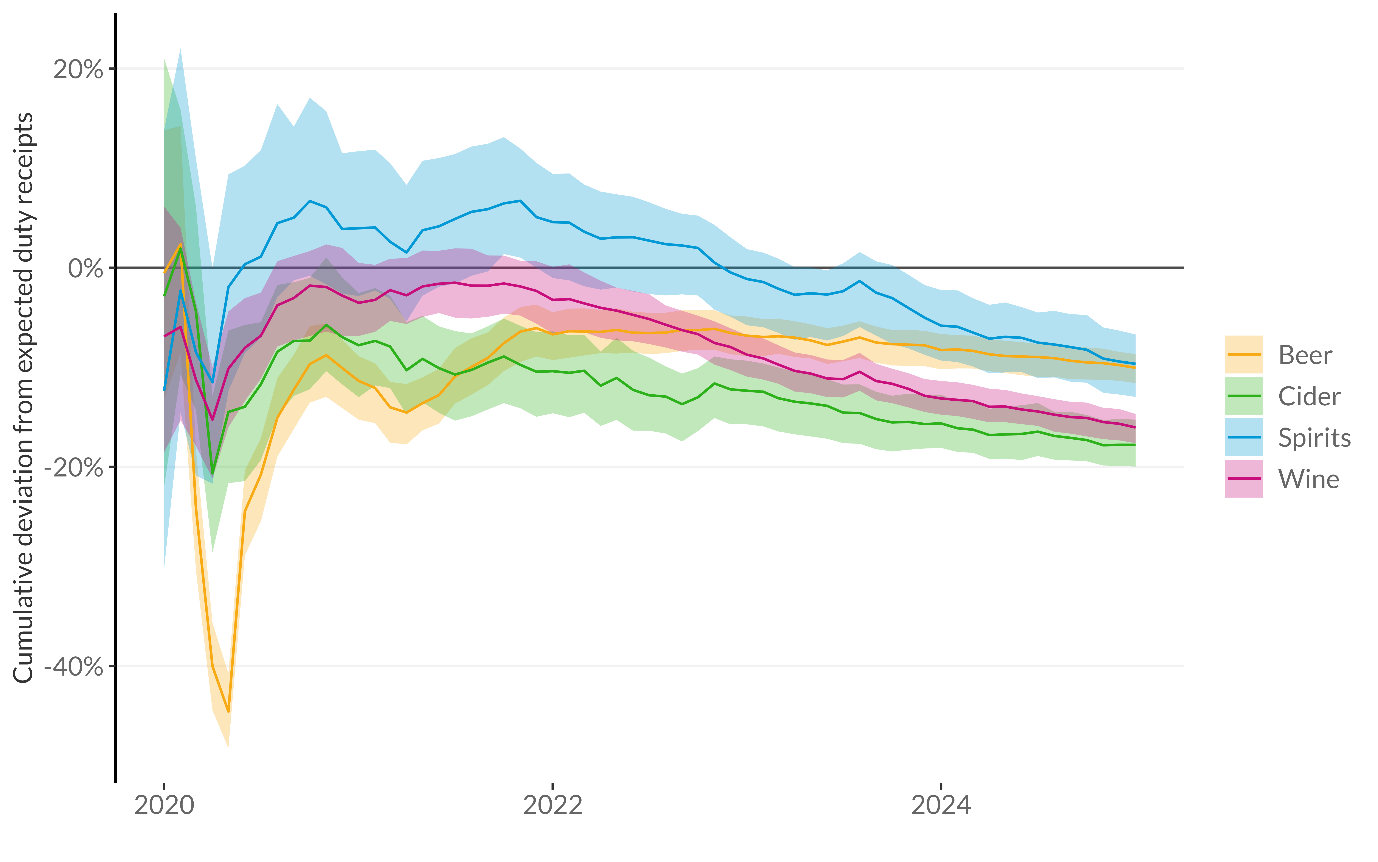
**

Figure A8 - Relative cumulative deviation from expected alcohol duty revenue in the UK by beverage type since January 2020 with 95% prediction interval

**Sensitivity analysis using training data up to December 2017 only**


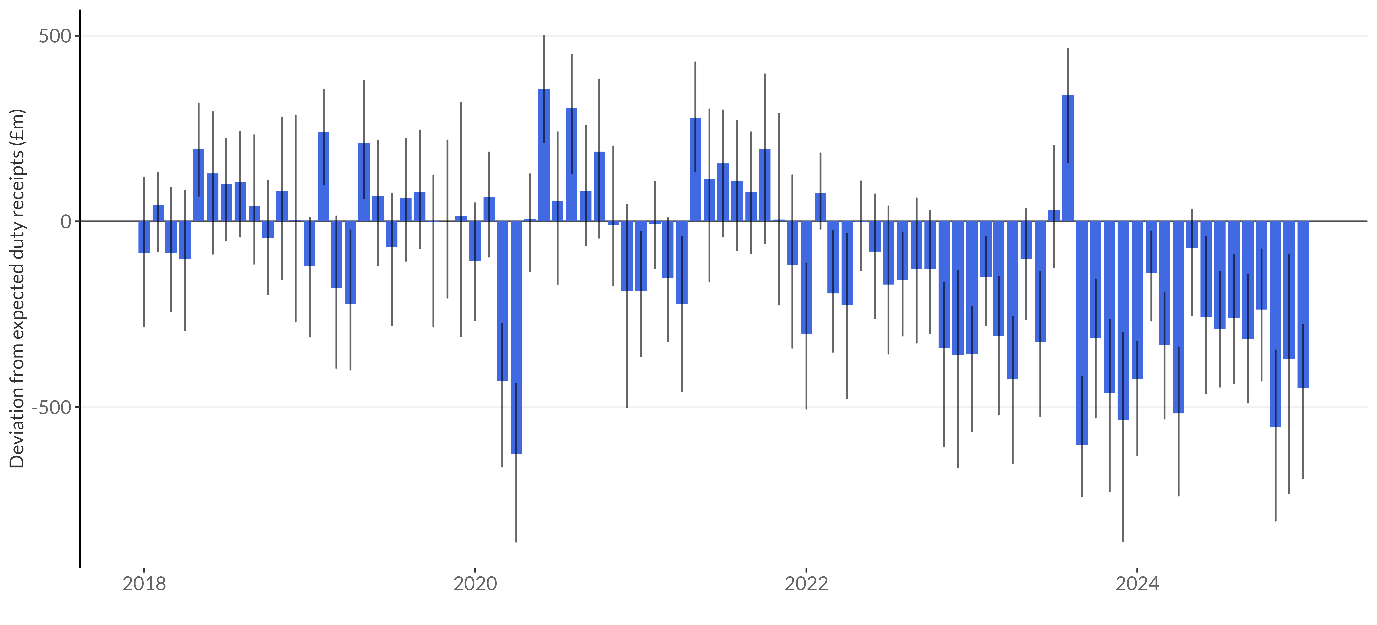


Figure A9 - Excess monthly duty revenue in the UK since January 2018 with 95% prediction intervals


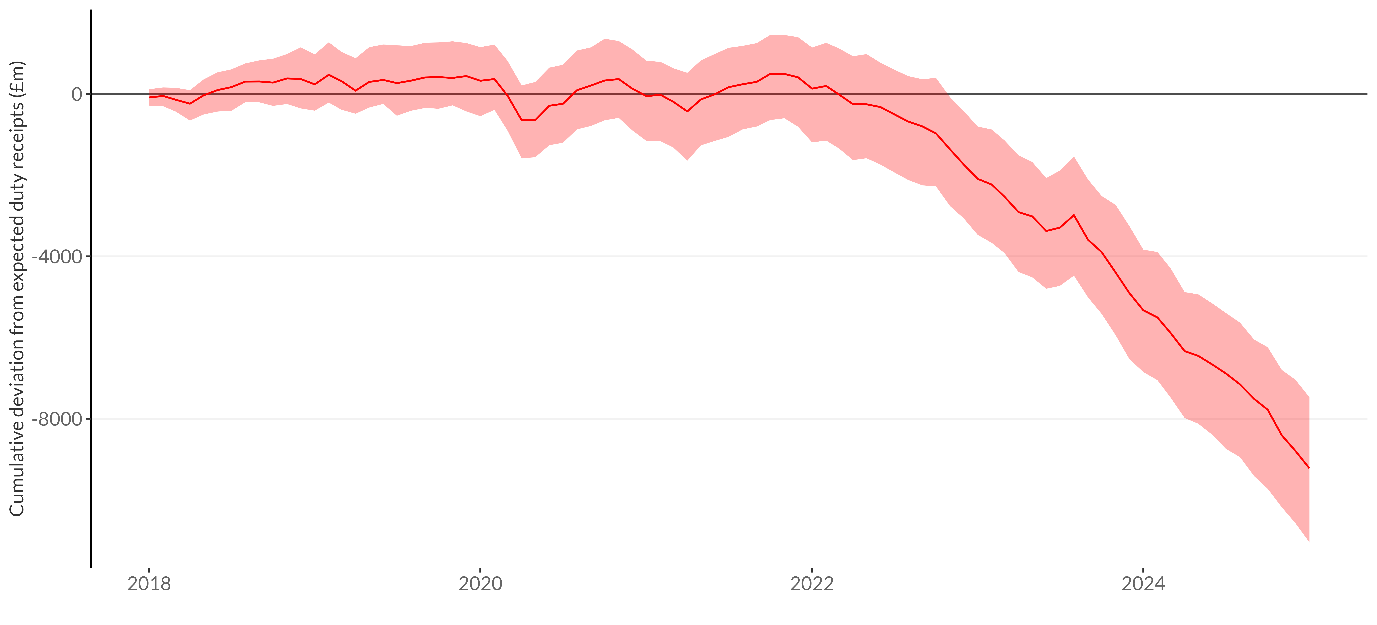


Figure A10 - Cumulative excess duty revenue since January 2018 with 95% prediction interval


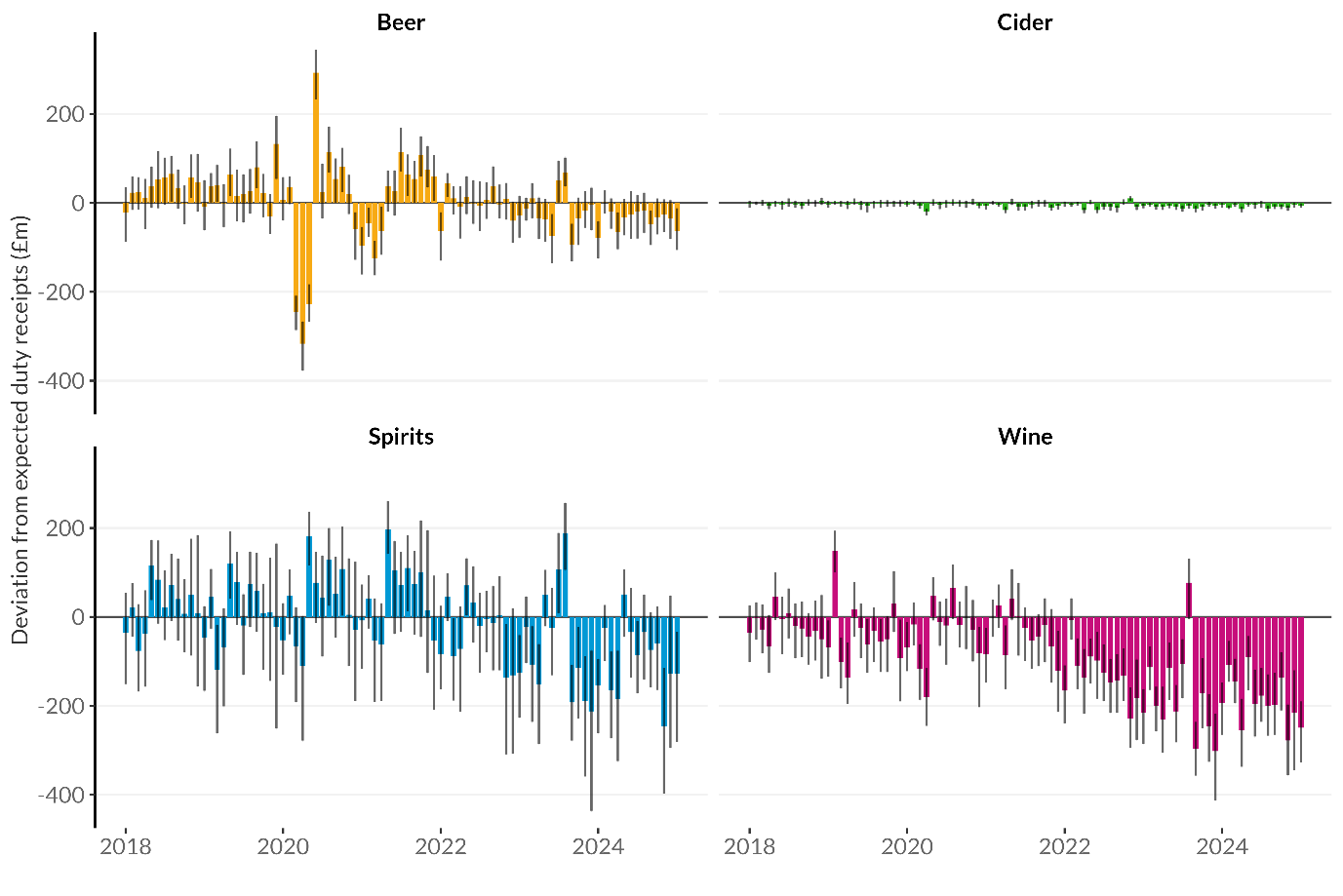


Figure A11 - Excess monthly duty revenue in the UK by beverage type since January 2018 with 95% prediction intervals


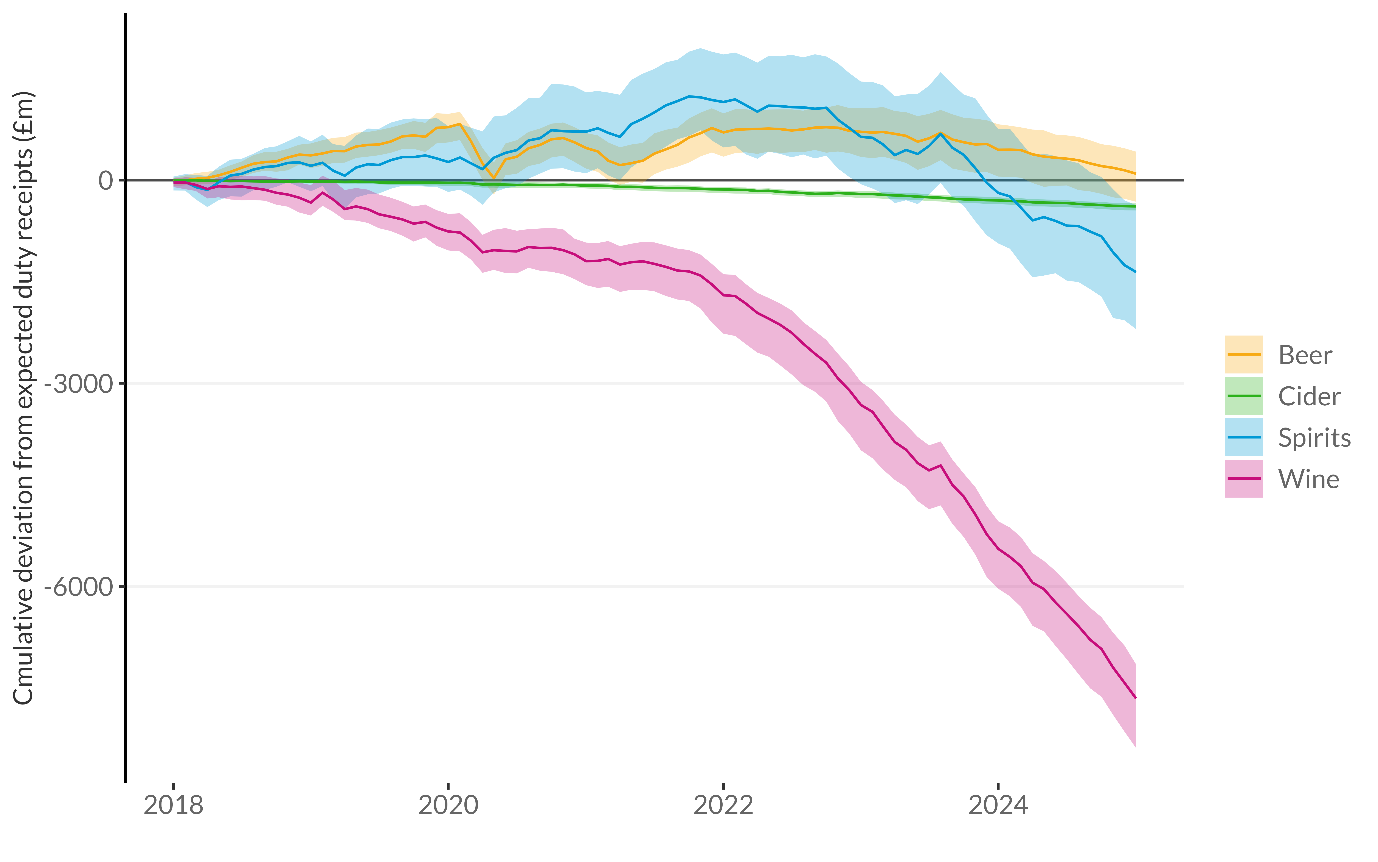


Figure A12 - Cumulative beverage-specific excess duty revenue since January 2018 with 95% prediction intervals
